# Interphase APC/C–Cdc20 inhibition by cyclin A2–Cdk2 ensures efficient mitotic entry

**DOI:** 10.1038/ncomms10975

**Published:** 2016-03-10

**Authors:** Jamin B. Hein, Jakob Nilsson

**Affiliations:** 1 The Novo Nordisk Foundation Center for Protein Research, Faculty of Health and Medical Sciences, University of Copenhagen, Blegdamsvej 3B, 2200 Copenhagen, Denmark

## Abstract

Proper cell-cycle progression requires tight temporal control of the Anaphase Promoting Complex/Cyclosome (APC/C), a large ubiquitin ligase that is activated by one of two co-activators, Cdh1 or Cdc20. APC/C and Cdc20 are already present during interphase but APC/C–Cdc20 regulation during this window of the cell cycle, if any, is unknown. Here we show that cyclin A2–Cdk2 binds and phosphorylates Cdc20 in interphase and this inhibits APC/C–Cdc20 activity. Preventing Cdc20 phosphorylation results in pre-mature activation of the APC/C–Cdc20 and several substrates, including cyclin B1 and A2, are destabilized which lengthens G2 and slows mitotic entry. Expressing non-degradable cyclin A2 but not cyclin B1 restores mitotic entry in these cells. We have thus uncovered a novel positive feedback loop centred on cyclin A2–Cdk2 inhibition of interphase APC/C–Cdc20 to allow further cyclin A2 accumulation and mitotic entry.

## Abstract (web summary)

The Anaphase Promoting Complex/Cyclosome (APC/C) and its co-activator Cdc20 regulate mitotic progression, but both are also present in interphase. Here Hein and Nilsson show that Cyclin A2–CDK2 phosphorylates Cdc20 to inhibit APC/C–Cdc20 activity during this cell cycle phase to promote mitotic entry.

## Graphical abstract

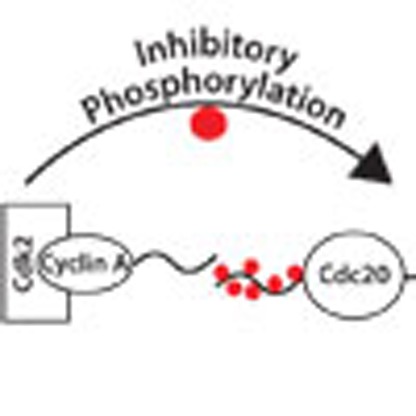

The Anaphase Promoting Complex/Cyclosome (APC/C), a large ubiquitin ligase, targets key cell-cycle regulators such as cyclin A2 (the major A-type cyclin in human somatic cells) and cyclin B1 for ubiquitin-mediated degradation hereby ensuring proper cell-cycle progression^1^,^2^. Cyclin A2 can activate both cyclin-dependent kinase (Cdk) 1 and 2, proline-directed kinases, and is an important regulator of mitotic entry by ensuring timely activation of the cyclin B1–Cdk1 complex^3^,^4^,^5^,^6^,^7^. The APC/C depends on one of two co-activators, Cdc20 or Cdh1, for binding destruction motifs in its substrates^1^. In addition to contributing to substrate binding the co-activators also directly activate the APC/C through an N-terminal C-box motif^8^,^9^. During mitosis, cyclin A2 and cyclin B1 are degraded in a precise temporal manner by the APC/C–Cdc20 complex^10^,^11^,^12^ and in the G1 phase by APC/C–Cdh1 (ref. 1). APC/C–Cdh1 is kept inactive in S and G2 by the Emi1 protein^13^ but whether mechanisms also exist to inhibit APC/C–Cdc20 in interphase, the time when cyclins need to accumulate to promote mitotic entry, is unknown. As full APC/C–Cdc20 activation requires the mitotic specific phosphorylation of the APC/C complex by cyclin B1–Cdk1 (refs 14, 15, 16, 17), any possible interphase APC/C–Cdc20 activity has been given little attention. *In vitro* studies have shown that interphase APC/C can be activated by Cdc20 although the complex might be less active compared to the mitotic form^11^,^16^,^18^,^19^. Cdc20 is inhibited by the spindle assembly checkpoint (SAC) during mitosis^20^ and in addition studies have shown that phosphorylation of Cdc20 by Cdk^21^,^22^,^23^ on multiple sites can decrease APC/C–Cdc20 activity by reducing APC/C–Cdc20 interaction. These previous studies have focused on Cdc20 phosphorylation in mitotic APC/C–Cdc20 regulation but radioactive labelling of Cdc20 suggested that it is phosphorylated already before mitosis^24^. The *in vivo* function of Cdc20 phosphorylation, its exact temporal control and the kinase(s) responsible are, however, largely unknown.

Here we show that cyclin A2–Cdk2 phosphorylates Cdc20 on sites close to the C-box in interphase. Cdc20 phosphorylation is required to inhibit the APC/C–Cdc20 complex to allow the accumulation of cyclins and hereby promote mitotic entry. We have thus uncovered a novel mechanism that inhibits Cdc20 before the activation of the SAC in mitosis.

## Results

### Cdc20 is phosphorylated prior to mitotic entry

**Figure 1:**
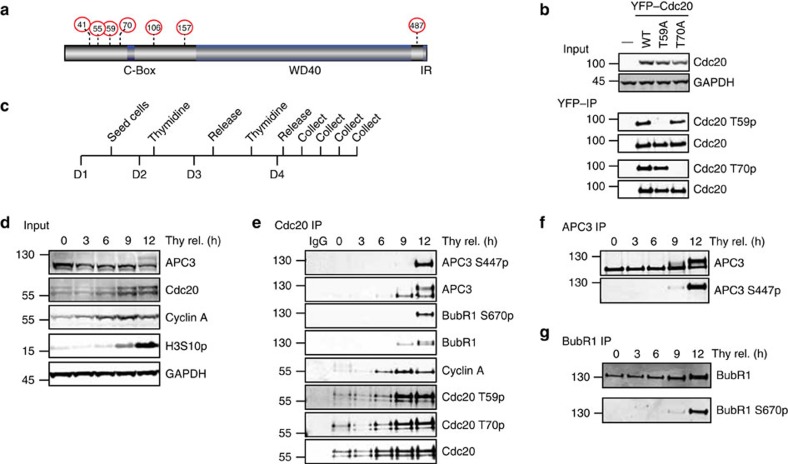
Cdc20 is phosphorylated in interphase. (**a**) Schematic of Cdc20 with known Cdk sites indicated by red circles and residue number. The C-Box motif, WD40 domain and IR tail is indicated as well. (**b**) YFP-tagged Cdc20, Cdc20 T59A or Cdc20 T70A was transiently expressed in HeLa cells and isolated using an YFP affinity resin from nocodazole-arrested cells harvested by mitotic shake-off. The purified complexes were analysed by SDS–PAGE and probed with polyclonal phospho-specific antibodies against Cdc20 T59 and Cdc20 T70, as well as a pan-specific Cdc20 antibody. (**c**) Synchronization protocol used to perform a double thymidine block and release experiment. D1–4 indicates day 1–4. (**d,e**) Western blot of input and Cdc20-purified samples from HeLa cells prepared as indicated in **c** and probed with the indicated antibodies. Representative of at least two independent experiments. (**f**,**g**) Western blot of APC3 and BubR1 purified samples prepared as in **c**. Representative of at least two independent experiments.

To understand the regulation and role of Cdc20 phosphorylation *in vivo*, we first generated phospho-specific antibodies against two conserved TP sites, Thr59 and Thr70 (T59 and T70), close to the C-Box of Cdc20 that have been reported to be phosphorylated by Cdks (Fig. 1a). Although Cdc20 is phosphorylated on multiple TP and SP sites, we focused on these two sites because previous work had shown these particular sites to be critical in regulating Cdc20 activity *in vitro*^21^. We first characterized the specificity of the antibodies by purifying yellow-fluorescent protein (YFP)-tagged Cdc20 wild type (WT), Cdc20 T59A and Cdc20 T70A from mitotic cells using YFP affinity resin. The T59 phospho-specific antibody did not recognize Cdc20 T59A while the T70 phospho-specific antibody did not recognize Cdc20 T70A (Fig. 1b). Thus, both antibodies recognize their specific phosphorylated residue. Importantly, if we treated immunopurified endogenous Cdc20 with lambda phosphatase (**λ**PPase) or depleted Cdc20 by RNA interference (RNAi), the phospho-specific antibodies no longer recognized a band migrating at the size of Cdc20 (Supplementary Fig. 1A,D). These tests validated the specificity of the antibodies but on whole-cell extracts they recognized several bands and we could therefore only use them on immunopurified Cdc20.

We next determined the timing of Cdc20 T59 and T70 phosphorylation and compared this with the timing of phosphorylation of the APC/C subunit APC3 on S447 and of BubR1 on S670, both of which are known mitotic Cdk1 sites^14^,^25^,^26^ (see Supplementary Fig. 1F for characterization of the APC3 antibody). We synchronized HeLa cells by using a double thymidine block protocol to arrest them in early S phase (Fig. 1c). Then, cells were released from the block and Cdc20 immunopurified at different times following the release using a monoclonal antibody recognizing Cdc20 just C-terminal to the C-box. The total level of Cdc20, as well as Cdc20 phosphorylation on T59 and T70 was measured using quantitative western blotting and compared with APC3 S447 and BubR1 S670 phosphorylation. This revealed a clear distinct temporal difference between Cdc20 phosphorylation and APC3 and BubR1 phosphorylation (Fig. 1d–g and Supplementary Fig. 3 for uncropped blots). While APC3 and BubR1 phosphorylation was almost absent until the 12-h time point, Cdc20 was phosphorylated earlier and peaked at 9 h after thymidine release. A similar trend in Cdc20 phosphorylation was observed in RPE1 and U2OS cells released from a single thymidine block (Supplementary Fig. 1B–E).

Our results clearly show that Cdc20 is phosphorylated on T59 and T70 in interphase and we speculated that Cdc20 phosphorylation might regulate APC/C–Cdc20 activity.

### Cyclin A2–Cdk2 phosphorylates Cdc20 before mitotic entry

**Figure 2:**
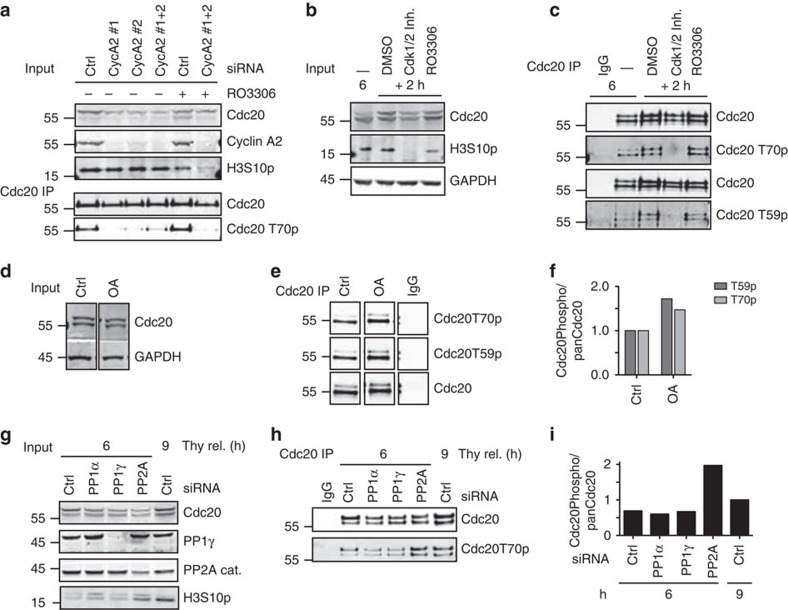
Cyclin A2–Cdk2 phosphorylates Cdc20. (**a**) Western blot of input and Cdc20-purified samples from HeLa cells treated with cyclin A2 siRNA (CycA2#1-2), synchronized with thymidine and collected 8 h after release from the thymidine block. Cdk1 inhibitor RO3306 was added 2 h before cells were collected. (**b,c**) Western blot of input and Cdc20-purified samples from HeLa cells synchronized with thymidine released for 6 h and then treated for 2 h with either Cdk1/2 Inhibitor III or RO3306. (**d**–**f**) Western blot of input and Cdc20-purified samples from HeLa cells synchronized with thymidine released for 6 h and then treated for 2 h with okadaic acid (OA). (**g**) HeLa cells were synchronized with a double thymidine block and depleted of PP1α, PP1γ or PP2A catalytic and scaffold subunit (PP2A). Six or nine hours after release from the second thymidine block, cells were collected. (**h**) Western blot of purified Cdc20 from experiment as in **g**. (**i**) Quantification of the signal obtained from the phospho-specific antibodies normalized to the pan Cdc20 signal. The phosphorylation level 9 h after thymidine release was set to 1. (**a**–**i**) are representative of at least two independent experiments.

Cyclin B1–Cdk1 can phosphorylate Cdc20 *in vitro* but is not activated until shortly before mitotic entry^5^, suggesting that a different proline-directed kinase was phosphorylating Cdc20 in interphase. Further supporting this idea is that cyclin B1 is cytoplasmic^27^ while Cdc20 is almost exclusively in the nucleus in interphase^24^. Interestingly, the peak in Cdc20 phosphorylation correlated with the time where binding to cyclin A2, a known interactor and substrate of Cdc20 (refs 11, 12, 28, 29), was at its maximum (Fig. 1e). As cyclin A2–Cdk2 has been reported to phosphorylate Cdc20 *in vitro*^21^,^29^ and is in the nucleus^27^, we investigated if this kinase phosphorylated Cdc20 *in vivo*. Indeed, RNAi of cyclin A2 using two distinct RNAi oligos strongly reduced Cdc20 phosphorylation on T70 8 h after release from thymidine (Fig. 2a). The total level of Cdc20 was also reduced when cyclin A2 was depleted and as discussed below we have found that Cdc20 phosphorylation stabilizes the protein. This decrease in Cdc20 levels on cyclin A2 depletion thus indirectly supports that cyclin A2 is required for phosphorylating Cdc20. Cyclin A2 can activate both Cdk1 and Cdk2 and we wanted to understand if a specific Cdk was responsible for phosphorylating Cdc20. Treatment of cells 6 h after release from a thymidine block with a Cdk1-specific inhibitor (RO3306) for 2 h did not affect Cdc20 phosphorylation showing that cyclin A2–Cdk1 is not responsible for Cdc20 phosphorylation (Fig. 2b,c). However a Cdk1/2 inhibitor (Cdk1/2 inhibitor III) blocked Cdc20 phosphorylation revealing that Cdk2 was required for Cdc20 phosphorylation (Fig. 2b,c). Our data thus shows that it is the cyclin A2–Cdk2 complex that phosphorylates Cdc20 prior to mitosis and the reported temporal increase in Cdk2 activity^30^ is in agreement with the Cdc20 phosphorylation changes we see. We observed that Cdc20 phosphorylation persists on mitotic entry (Fig. 1e) despite cyclin A2 being degraded at mitotic entry^11^,^12^. Indeed Cdc20 phosphorylation persisted in cells arrested for a prolonged time in mitosis by nocodazole and this depends on Cdk1 activity arguing that cyclin B1–Cdk1 keeps Cdc20 phosphorylated in mitosis (Supplementary Fig. 1G).

Interestingly, addition of okadaic acid (OA) to interphase cells increased the level of Cdc20 T59 and T70 phosphorylation, suggesting that an OA-sensitive phosphatase antagonizes cyclin A2–Cdk2-mediated phosphorylation of Cdc20 (Fig. 2d–f). The combined depletion of the catalytic and scaffold subunits of the protein phosphatase 2A^31^ (PP2A) increased Cdc20 T70 phosphorylation 6 h after release from a thymidine block (Fig. 2g–i). This suggests that Cdc20 phosphorylation is dynamic and points to PP2A as the phosphatase antagonizing cyclin A2–Cdk2 phosphorylation of Cdc20 during interphase.

### Cdc20 phosphorylation promotes mitotic entry

**Figure 3:**
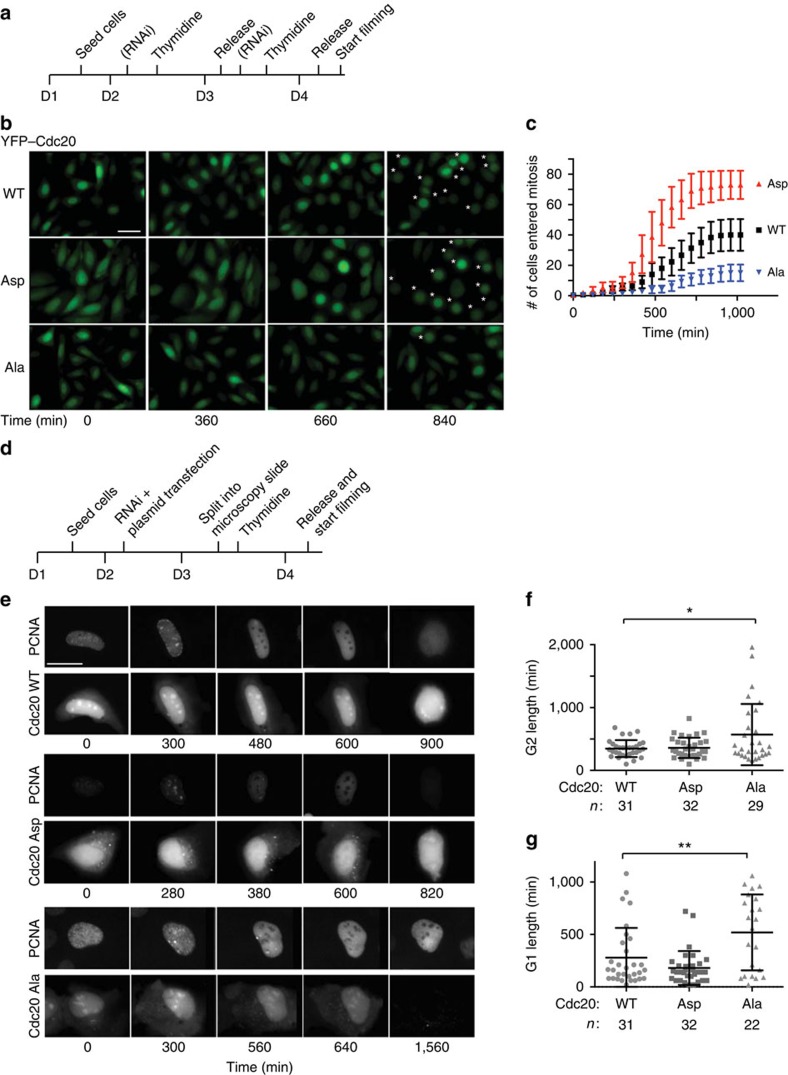
Cdc20 phosphorylation promotes mitotic entry. (**a**) Outline of the set-up of time-lapse microscopy experiment with stable HeLa-FRT cells. D1–4 indicate day 1–4. (**b**) Representative images of HeLa cells stably expressing the indicated YFP-tagged Cdc20 as they progress into mitosis following release from a double thymidine block into nocodazole. Star indicates cells in mitosis. Scale bar, 40 μm. (**c**) Cumulative frequency plot of cells entering mitosis as counted from experiments outlined in **a**. The time indicates the time the cells were filmed (*T*=0 is ∼2 h after release from double thymidine block) and error bars are s.e.m. Representative of three independent experiments. (**d**) Outline of the set-up of time-lapse microscopy experiment with U2OS cells stably expressing GFP-tagged PCNA and transiently transfected with the indicated mCherry–Cdc20 constructs. (**e**) Representative images of U2OS cells from experiments outlined in **d** with the GFP signal (PCNA) and mCherry signal (Cdc20) shown at the indicated time after the filming started. The filming was started ∼2 h after release from the thymidine block. Scale bar, 40 μm. (**f**) Quantification of the time cells spent in G2 by measuring the time when PCNA foci dissolves until nuclear envelope breakdown (NEBD). A Mann–Whitney test was used for statistical comparison (*P≤0.05). *n*: number of cells analysed from three independent experiments. (**g**) Quantification of the time cells spent in G1 by measuring the time from mitotic exit to the appearance of PCNA foci. A Mann–Whitney test was used for statistical comparison (**P≤0.005). *n*: number of cells analysed from three independent experiments. For **f,g,** several Cdc20^Ala^-expressing cells were arrested for the duration of filming and thus the effect on G1 and G2 length is like underestimated for this Cdc20 mutant.

Although we can only measure the phosphorylation of Cdc20 on T59 and T70, we anticipate that cyclin A2–Cdk2 phosphorylates all TP and SP sites in Cdc20 as most of them confer to the Cdk consensus sequence (S/T-P-X-R/K). Work from the Yamano lab^21^ has shown that phosphorylation of multiple sites on Cdc20 is required to fully inhibit Cdc20 activity in egg extracts. Our initial analysis of a Cdc20 mutant with Thr55, Thr59 and Thr70 mutated to alanine did not exhibit any cellular phenotypes. Therefore to understand the *in vivo* role of Cdc20 phosphorylation on Cdk sites, we decided to use Cdc20^Ala^ and Cdc20^Asp^ that are previously described mutants where all known and putative Cdk sites (Ser41, Thr55, Thr59, Thr70, Thr106, Thr157 and Ser487) are mutated to either Ala or Asp^16^. Cdc20^Ala^ should mimic non-phosphorylated Cdc20 while Cdc20^Asp^ should mimic fully phosphorylated Cdc20. First we generated isogenic stable HeLa cell lines expressing inducible RNAi resistant YFP-tagged Cdc20 proteins. Cells were synchronized with a double thymidine block, treated twice with an RNAi oligo that efficiently depletes Cdc20 and YFP–Cdc20 protein was induced by adding doxycycline to the media (Fig. 3a). We observed that YFP–Cdc20^Asp^ is more stable than YFP–Cdc20^WT^ and YFP–Cdc20^Ala^ and in several experiments YFP–Cdc20^Ala^ was degraded in cells (see, for example, Figs 3e and 6c). Therefore different concentrations of doxycycline were used to obtain similar levels of expression. Cells were followed 2 h after release from the last thymidine block by live cell imaging. YFP–Cdc20^WT^-expressing cells entered mitosis during the 18 h of filming while fewer cells expressing YFP–Cdc20^Ala^ did so (Fig. 3b,c, Supplementary Movies 1–3). In contrast, more YFP–Cdc20^Asp^-expressing cells entered mitosis than YFP–Cdc20^WT^-expressing cells. There was no correlation between the few cells entering mitosis and the expression level of YFP–Cdc20^Ala^ and similar results were obtained using stable isogenic U2OS cells (Supplementary Fig. 2A,B). A different Cdc20 mutant where phosphorylation of the five Cdk sites in the N-terminal tail was prevented by mutating the proline residue of the Cdk sites to alanine displayed a similar phenotype to Cdc20^Ala^ further supporting that Cdc20 phosphorylation was critical for mitotic entry (Supplementary Fig. 2C).

We made whole-cell extracts with 3-h intervals following thymidine release and monitored auto-phosphorylation on cyclin B1 S126, a site that is phosphorylated just prior to mitotic entry^32^. In agreement with the live cell experiments, S126 phosphorylation on cyclin B1 accumulated to lower levels in the YFP–Cdc20^Ala^-expressing cells and did not display a similar peak as in the YFP–Cdc20^WT^- and YFP–Cdc20^Asp^-expressing cells at 12 h after release (Supplementary Fig. 2D,E). Next, we determined if the mitotic entry defect in YFP–Cdc20^Ala^-expressing cells was related to lack of inhibition of Cdc20 by interphase SAC inhibitory complexes^33^,^34^. We monitored mitotic entry in a cell line stably expressing YFP–Cdc20 R132A that cannot bind the Mad2 checkpoint protein^35^,^36^. However, cells entered mitosis normally in YFP–Cdc20 R132A-expressing cells (Supplementary Fig. 2F), indicating that the observed delay in mitotic entry is unrelated to the lack of SAC inhibition of YFP–Cdc20^Ala^.

Since our data showed that Cdc20 was phosphorylated more strongly in G2 than in S, we wanted to investigate if preventing Cdc20 phosphorylation affected the duration of G2. A U2OS cell line stably expressing green fluorescent protein (GFP–PCNA) was co-transfected with mCherry-tagged Cdc20 constructs and the Cdc20 RNAi oligo and monitored by time-lapse microscopy (Fig. 3d). The length from the disappearance of PCNA foci to nuclear envelope breakdown was used as a measure of G2 duration (Fig. 3e,f). This revealed that indeed expression of mCherry–Cdc20^Ala^ lengthened G2 in agreement with the observed effect on mitotic entry. We cannot rule out an effect on S phase progression in Cdc20^Ala^-expressing cells but we observed no difference in EdU incorporation 3 h after release from the last thymidine block arguing that initiation of S phase is similar in the cell lines (Supplementary Fig. 2G and Supplementary Methods). The mCherry–Cdc20^Ala^-expressing cells that did enter mitosis progressed normally through this and then displayed a prolonged arrest in the subsequent G1 (Fig. 3g). This potentially reflects a low level of cyclin A2–Cdk2 activity in G2 (see below) that affects the length of the subsequent G1 as shown by the Meyer lab^30^. Combined, these experiments show that preventing phosphorylation of Cdc20 has severe consequences on several aspects of cell-cycle progression.

### Cdc20 phosphorylation inhibits interphase APC/C binding

**Figure 4:**
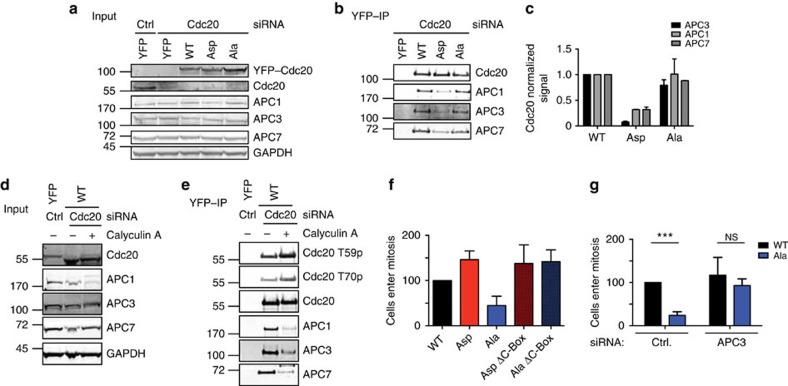
Cdc20 phosphorylation prevents interphase APC/C binding. (**a,b**) Stable HeLa cell lines expressing the indicated YFP-tagged Cdc20 proteins were depleted of endogenous Cdc20 and 8 h after release from a thymidine block YFP–Cdc20 complexes were purified. The samples were analysed by SDS–PAGE and probed for APC1, APC3 and APC7 and this was quantified by LI-COR technology. (**c**) Bar diagram shows the median and range from two (for APC1 and 7) or three (for APC3) independent experiments. Signals were normalized to Cdc20 and Cdc20 WT. (**d,e**) As in **a,b** but YFP-tagged Cdc20 WT cells were treated after 7.5 h for 30 min with Calyculin A and then collected. Representative of two independent experiments. (**f**) Stable HeLa cell lines expressing the indicated YFP-tagged Cdc20 proteins were synchronized as outlined in Fig. 3a and following release from the last thymidine block followed by time-lapse microscopy. The bar graph shows cells entering mitosis after normalization to Cdc20 WT-expressing cells. Average of two independent experiments (at least 127 cells for each condition) with range indicated by the lines. (**g**) Stable HeLa cell lines expressing YFP–Cdc20^WT^ or YFP–Cdc20^Ala^ were synchronized as outlined in Fig. 3a and after release from the last thymidine block mitotic entry was monitored by time-lapse microscopy. In these experiments, Cdc20 was not depleted but cells instead treated with a control siRNA (luciferase (ctrl)) or a siRNA-targeting APC3. Average of three experiments (at least 312 cells in each condition) is indicated in the bar graph with s.d. indicated by the line. An unpaired *t*-test without assuming equal s.d. was used for statistical comparison (***P<0.0001; NS, non-significant).

In the purifications of Cdc20, we observed an interaction between the APC/C and Cdc20 prior to phosphorylation of the APC/C (Fig. 1e), suggesting that some low level of APC/C–Cdc20 activity might exist before mitosis. Given that phosphorylation of Cdc20 has been shown to negatively regulate its interaction with the APC/C *in vitro* and in extracts^21^, we wanted to determine if the same was the case during interphase in human cells. First, we compared the ability of YFP–Cdc20^Ala^ and YFP–Cdc20^Asp^ to interact with interphase APC/C. The stable HeLa cell lines were depleted of endogenous Cdc20 and the cell population enriched for interphase cells by removing mitotic cells by shake-off. YFP–Cdc20 complexes were isolated 8 h after thymidine release using YFP affinity resin and probed for APC/C subunits. Indeed, both YFP–Cdc20^WT^ and YFP–Cdc20^Ala^ co-purified APC/C subunits while YFP–Cdc20^Asp^ was impaired in its ability to bind the APC/C (Fig. 4a–c). APC3 displays a characteristic retardation on SDS–PAGE during mitosis but this was not observed with the co-purifying APC3 in these samples. This shows that phosphorylation of Cdc20 can negatively regulate the interaction with the APC/C in interphase. The fact that YFP–Cdc20^WT^ and YFP–Cdc20^Ala^ co-purify similar amounts of APC/C could reflect that only a fraction of Cdc20 is phosphorylated in G2 or that YFP–Cdc20^WT^ is dephosphorylated during purification. In a separate experiment, we treated YFP–Cdc20^WT^-expressing interphase cells with the protein phosphatase inhibitor Calyculin A for 30 min. This strongly increased Cdc20 phosphorylation and reduced APC/C binding (Fig. 4d,e). Importantly APC3 was still in its unphosphorylated form in these experiments further supporting that Cdc20 phosphorylation blocks it's binding to interphase APC/C.

Next, we addressed if the defect of mitotic entry observed in cells expressing YFP–Cdc20^Ala^ related to the role of Cdc20 in activating the APC/C. To do this, we mutated three conserved residues in the C-Box (amino acids 78–80 to alanine) in YFP–Cdc20^Ala^ and YFP–Cdc20^Asp^, which we previously have shown to prevent mitotic APC/C–Cdc20 activity^37^. We then monitored the ability of stable HeLa cell lines to enter mitosis following release from a thymidine block by live cell microscopy. The delay in entering mitosis observed in YFP–Cdc20^Ala^-expressing cells was not observed in YFP–Cdc20^Ala^ ΔC-Box-expressing cells and cyclin B1 S126 phosphorylation displayed a profile similar to YFP–Cdc20^WT^-expressing cells (Fig. 4f and Supplementary Fig. 2D,E). This argues that the delay in mitotic entry on expression of YFP–Cdc20^Ala^ requires that it can activate the APC/C. This was further supported by directly inhibiting the APC/C in the stable YFP–Cdc20^Ala^ cell line. When the APC3 subunit was depleted in this cell line then cells entered mitosis as efficiently as YFP–Cdc20^WT^-expressing cells (Fig. 4g).

### APC/C–Cdc20 can degrade substrates in interphase

**Figure 5:**
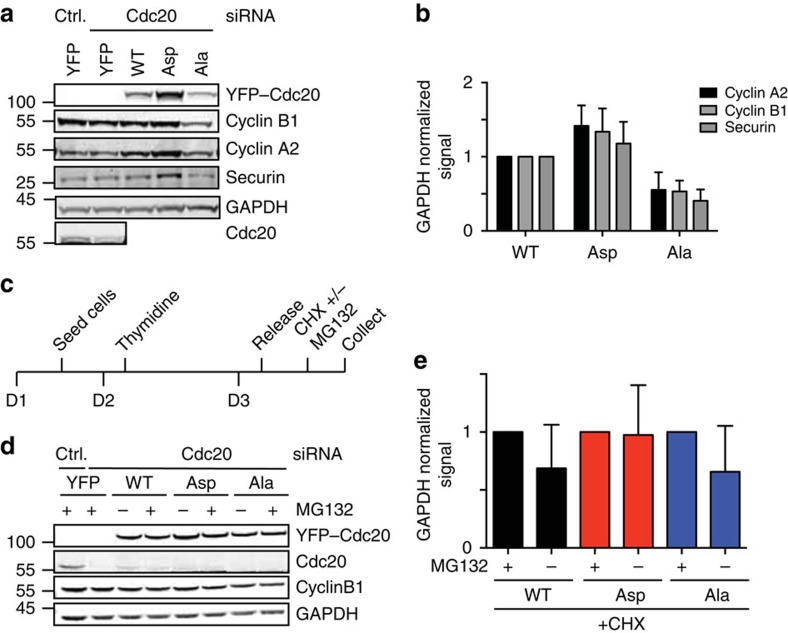
The APC/C–Cdc20 is active in interphase. (**a**) Stable HeLa cell lines expressing the indicated YFP-tagged Cdc20 proteins were depleted of endogenous Cdc20, released from one 20-h thymidine block and cells were collected 9 h after the release. Samples were analysed by SDS–PAGE and the level of the indicated proteins was determined by quantitative western blot. (**b**) Quantification of six experiments similar to **a**. Western blot fluorescence intensity signal was normalized to either Vinculin or GAPDH and the level in YFP–Cdc20^WT^-expressing cells was set to 1. The average and s.d. is indicated. (**c**) Outline of synchronization and experimental set-up to look at protein turnover. (**d**) Cells were depleted of endogenous Cdc20, released from a 20-h thymidine block and 6 h after release treated with cycloheximide (CHX) and with MG132 where indicated (+) for 80 min. Total cell extract was analysed by western blot and probed for the indicated proteins. (**e**) Quantification of five experiments as in **d**. Western blot fluorescence intensity signal was normalized to GAPDH and Cdc20^WT^ set to 1. The bar diagram shows the average and s.d.

Our data suggested that there is some APC/C–Cdc20 activity prior to mitotic entry that is inhibited by cyclin A2–Cdk2 phosphorylation of Cdc20 and therefore we determined the interphase levels of a number of known APC/C–Cdc20 substrates. Cells were synchronized as outlined (Fig. 5c) and harvested at 9 h and total cell extracts were prepared. Using quantitative western blot the protein levels of cyclin B1, cyclin A2 and securin normalized to GAPDH were specifically reduced in YFP–Cdc20^Ala^-expressing cells (Fig. 5a,b). These results suggested that the APC/C was prematurely activated in interphase on expression of YFP–Cdc20^Ala^ although a change in cell-cycle distribution could also account for the difference. To test directly whether APC/C–Cdc20 was active in interphase and this activity was regulated by Cdc20 phosphorylation, we monitored cyclin B1 stability 6 h after release from the thymidine block. This time point was chosen to ensure that cells did not enter mitosis during the course of the experiment. The different cell lines were treated with cycloheximide for 80 min either in the presence or absence of a proteasome inhibitor (MG132) and the levels of cyclin B1 in cell extracts determined by quantitative western blot (Fig. 5c–e). In cells expressing YFP–Cdc20^WT^ or YFP–Cdc20^Ala^, cyclin B1 levels dropped by 30–40% in the absence of MG132 while in cells expressing YFP–Cdc20^Asp^ the cyclin B1 levels remained constant. The fact that cyclin B1 was destabilized in both YFP–Cdc20^WT^ and YFP–Cdc20^Ala^ cells is in agreement with our observation that between 6 and 8 h after thymidine release full Cdc20 phosphorylation has not yet been achieved. The result clearly supports that there is APC/C–Cdc20 activity in interphase that can be inhibited by phosphorylation of Cdc20. However, the 30–40% reduction in cyclin B1 during 80 min of cycloheximide incubation is less compared with the mitotic rate of cyclin B1 degradation.

**Figure 6:**
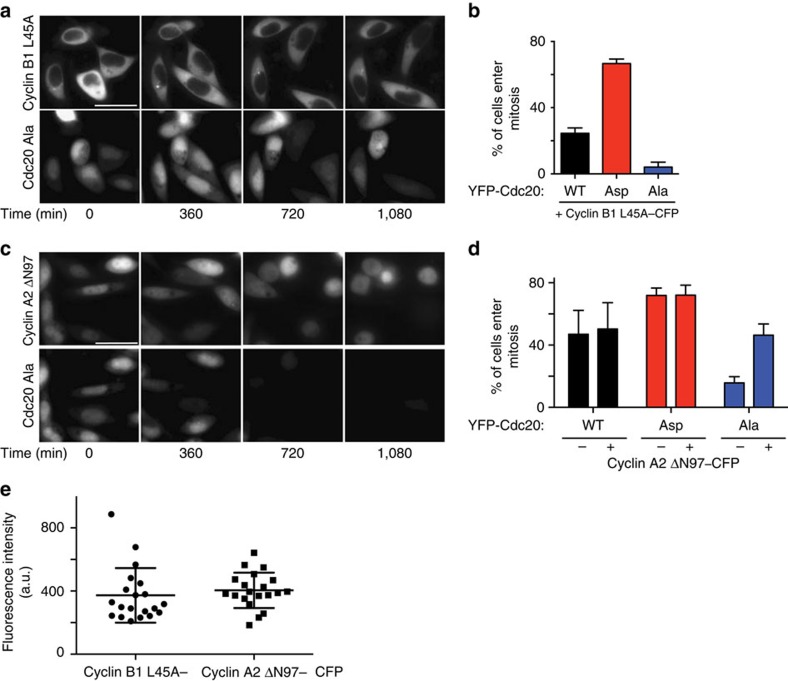
Non-degradable cyclin A2 restores mitotic entry. (**a,b**) HeLa cells stably expressing the indicated YFP-tagged Cdc20 proteins were synchronized as outlined in Fig. 3a and transfected with cyclin B1 L45A –CFP at 24 h before filming and monitored by time-lapse microscopy. Filming was started 2 h after release from the last thymidine block. Quantification of experiment shows percentage of cyclin B1 L45A–CFP-transfected cells, which enter mitosis during the course of the experiment. Mean of three experiments (at least 300 cells analysed in each condition) with error bars showing s.d. Representative still images from YFP–Cdc20^Ala^ is shown. Scale bar, 40 μm. (**c**,**d**) Quantification of live cell experiment similar to **a**,**b**. Percentage of cyclin A2 ΔN97–CFP-transfected cells, which enter mitosis during the course of the experiment compared with non-transfected cells. Mean of three independent experiments (at least 200 cells analysed in each condition) with error bars showing s.d. (**e**) The mean fluorescence intensity of cyclin B1 L45A–CFP and cyclin A2 ΔN97–CFP was analyzed in 20 cells from the experiments in **a**–**d** and plotted.

To determine if the mitotic entry defect in YFP–Cdc20^Ala^-expressing cells was due to lowered cyclin levels, we expressed a non-degradable cyclin B1 tagged with cyan fluorescent protein (cyclin B1 L45A–CFP^38^) or non-degradable cyclin A2 (cyclin A2 ΔN97–CFP^12^) in the different cell lines. Although cyclin B1 L45A was clearly active as it induced a mitotic arrest in YFP–Cdc20^WT^ cells it did not rescue mitotic entry in YFP–Cdc20^Ala^-expressing cells (Fig. 6a,b). However, expression of cyclin A2 ΔN97–CFP at a similar fluorescent intensity as cyclin B1 L45A allowed YFP–Cdc20^Ala^ cells to enter mitosis normally (Fig. 6c–e), which fits with previous observations that cyclin A2 has a more critical role in mitotic entry than cyclin B1 (ref. 3).

## Discussion

**Figure 7:**
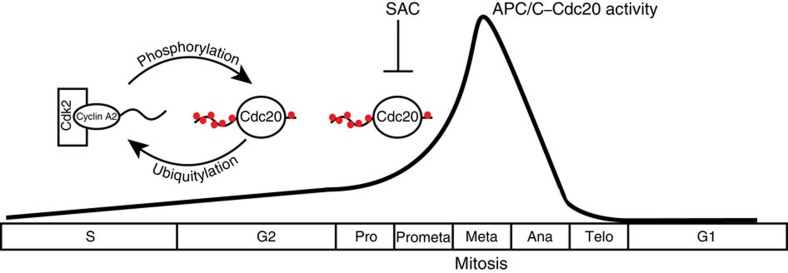
Regulation of APC/C–Cdc20 activity. A schematic of the regulation of APC/C–Cdc20 activity in interphase by cyclin A2–Cdk2 phosphorylation of Cdc20. On mitotic entry, cyclin A2 is degraded and the SAC inhibits APC/C–Cdc20 instead. The black line indicates the hypothetical strength of APC/C–Cdc20 activity during the cell cycle.

The APC/C complex is a major regulator of the cell cycle and its activity is tightly controlled. Here we show for the first time that APC/C–Cdc20 has activity in interphase and that phosphorylation of Cdc20 by cyclin A2–Cdk2 is required to repress this activity to ensure efficient mitotic entry (Fig. 7). The steady increase we see in Cdc20 phosphorylation as cells progress through S and G2 fits well with the reported increase in cyclin A2–Cdk2 activity monitored using a biosensor^30^. Cdc20 phosphorylation on all Cdk sites prevents binding to the APC/C but we envision that some of the sites might also be inhibitory without blocking APC/C binding. Whether the different Cdk sites are phosphorylated to different extents requires further investigations.

Previous work has clearly shown that phosphorylation of the APC/C by cyclin B1–Cdk1 in mitosis stimulates APC/C–Cdc20 activity^15^,^16^,^17^ providing an additional mechanism of restricting APC/C–Cdc20 activity to mitosis. We estimate that APC/C–Cdc20 is around five times less active in interphase compared with mitosis based on the fact that we only observe a 30–40% reduction in cyclin B1 levels in 80 min (Fig. 5), while the half-life of cyclin B1 is ∼20 min at metaphase in HeLa cells^10^. Given the longer duration of interphase compared with mitosis, it is not surprising that inhibition of this interphase APC/C–Cdc20 activity is critical for the cell cycle.

The interphase APC/C–Cdc20 activity could either be due to unphosphorylated APC/C–Cdc20 having some activity *in vivo* or that a small amount of Cdk1 phosphorylated APC/C exist in interphase. Indeed, the threshold of Cdk1 activity required for maximum APC/C–Cdc20 activity is lower than that required for full APC3 phosphorylation^39^, suggesting that even low levels of Cdk1 activity might be sufficient to trigger APC/C–Cdc20 activity. We do not know if there is a particular period in interphase where repression of APC/C–Cdc20 activity is critical but we favour that this is in late G2/prophase, where we observe a high level of Cdc20 phosphorylation and where cyclin B1–Cdk1 activity starts to rise^5^,^40^. This would be in agreement with our observation that between 6 and 8 h after thymidine release, Cdc20^WT^ is not repressed by phosphorylation (Fig. 5c–e).

Our work also uncovers a positive feedback loop where cyclin A2 ensures its own stability to accumulate sufficient cyclin A2–Cdk2 levels to promote mitotic entry. In addition, the phosphorylation of Cdc20 by cyclin A2–Cdk2 stabilizes the protein in interphase thus contributing to Cdc20 accumulation. Why Cdc20 phosphorylation stabilizes the protein is not clear but since Cdc20^Ala^ is unstable while Cdc20^Ala^ ΔC-box is stable it suggests that Cdc20 might autoubiquitinate itself in interphase. We favour that the phosphorylation of Cdc20 blocks this autoubiquitination, leading to Cdc20 stabilization.

Interestingly, on mitotic entry cyclin A2 is rapidly degraded but the SAC becomes active showing that two distinct mechanisms act in consecutive order to ensure APC/C–Cdc20 inhibition. Cdc20 phosphorylation persists as cells enter mitosis because cyclin B1–Cdk1 is now active and has access to Cdc20. This raises the question of how cyclin A2 can be degraded at mitotic entry when Cdc20 is phosphorylated? We favour that the higher APC/C–Cdc20 activity in mitosis cannot be fully inhibited by Cdc20 phosphorylation allowing cyclin A2 degradation. This is possibly why the SAC uses a different and more potent mechanism of inhibiting Cdc20 during mitosis.

## Methods

### Cloning and generation of stable cell lines

Full-length RNAi-resistant Cdc20 was amplified by PCR and cloned into the BamHI and NotI sites of pcDNA5/FRT/TO 3*FLAG–YFP. Cdc20 Asp and Ala constructs (gift from Jan-Michael Peters) and Cdc20 5P (Geneart synthesis) were cloned into the BamHI/BglII and NotI sites of pcDNA5/FRT/TO 3*FLAG–YFP and made resistant to the RNAi oligo by whole-plasmid PCR (5′- CCAGCCGGAAAACTTGTAGATACATTCCTTCC -3′). The T59A and T70A mutations were introduced by whole-plasmid PCR into Cdc20 WT plasmid (5′- GACTCCGGGCCGAGCTCCTGGCAAATC -3′ and 5′- CAGGTTTGCTAGGAGCGGTCTGAACCTTG -3′). The mutation of three residues of the C-Box in Cdc20 constructs was obtained by whole-plasmid PCR using the following primer: 5′- GCAAACCTGGCGGTATCCCCCATCGCAGTG -3′ and complementary reverse primer. Cdc20 WT, Asp and Ala contructs were subcloned with BamHI/BglII and NotI into pcDNA5 mCherry vector. The generation of stable HeLa cell lines expressing constructs under the control of a doxycyline-inducible promotor was done as previously described^37^.

### Antibodies

The following antibodies were used at the indicated dilutions for western blot. Cdc20 mouse monoclonal (sc-13162, 1:1,000, Santa Cruz), Cdc20 rabbit polyclonal (A301-180A-1, 1:1,000, Bethyl Laboratories), GAPDH rabbit polyclonal (sc-25778, 1:500, Santa Cruz), APC1 rabbit polyclonal (A301-653A-1, 1:500, Bethyl Laboratories), APC3 mouse monoclonal (610454, 1:500, BD Biosciences), APC7 rabbit polyclonal (A302-551A-1, 1:500, Bethyl Laboratories), Cdc20 T70 and T59 phosphorylation specific antibodies (raised against CSKVQT(Tp)PSKPG and CRTPGR(Tp)PGKSS, respectively; 1:500 and 1:250, Moravian Biotechnology). APC3 S447p (raised against CGKISTI(Tp)PQIQAF; 1:500, Moravian Biotechnology), BubR1 S670p (raised against TLSIKKL(Sp)PIIED,1:500, 21st Century), cyclin B1 S126p (Gift from Jonathon Pines, 1:500), Anti-phospho Histone H3 (Ser 10) rabbit polyclonal (06-570, 1:1,000, Millipore), cyclin B1 mouse monoclonal (554177, 1:1,000, BD Pharmingen), cyclin A rabbit polyclonal (sc-751, 1:1,000, Santa Cruz), Vinculin mouse monoclonal (V9131, 1:10,000, Sigma), Securin mouse monoclonal (K0090-3, 1:500, MBL), PP1 gamma rabbit polyclonal (A300-906A, Bethyl, 1:500), PP2A catalytic subunit mouse monoclonal (05-421, Millipore, 1:2,500). BubR1 mouse monoclonal (raised against TPR domain, 1:500, Biotech Research and Innovation Center, Copenhagen).

### RNAi and Plasmid transfection

Endogenous Cdc20 was depleted using Lipofectamine RNAi max from Life Technologies according to manufacturer's instructions. Cdc20 siRNA (5′- CGGAAGACCUGCCGUUACAUU -3′) was obtained from Sigma or Dharmacon and used at a concentration of 100–125 nM (Sigma) and 50 nM (Dharmacon). APC3 siRNA (5′- GGAAAUAGCCGAGAGGUAA -3′) was obtained from Sigma and used at a concentration of 100 nM. Cyclin A2 silencer select siRNA #1 (5′- CUAUGGACAUGUCAAUUGU -3′) and #2 (5′- GAUAUACCCUGGAAAGUCU -3′) were obtained from Life Technologies and used at a concentration of 10 nM. PP1α siRNA (5′- GCAAGAGACGCUACAACAU -3′ and 5′- GCAGUCUAUGGAGCAGAUU -3′), PP1γ siRNA (5′- GCUUAAAGUCUCGUGAAAU -3′ and 5′- GCCUAUCCUACUAGAACUU -3′), PP2A catalytic subunit and PP2A scaffold siRNA according to the study by Schmitz *et al*^31^ were obtained from Sigma and used at 50 nM. In biochemical experiments, cells were treated for 24–36 h prior to harvesting. In live cell imaging experiments, cells were treated with siRNA for 24 h prior to imaging. In experiments with U2OS cells stably expressing GFP–PCNA^41^, cells were transfected 48 h prior to imaging in six-well dishes using FuGene6 with 2 μg DNA and 75 nM siRNA. In live cell experiments with non-degradable cyclin B1 (pcDNA5 FRT/TO cyclin B1 L45A–CFP) and cyclin A2 (pcDNA5 FRT/TO ΔN97-cyclin A2–CFP) (Gift from Jonathan Pines), stable HeLa Cdc20 cell lines were transfected with plasmid for 5 h between two thymidine blocks ∼24 h prior to imaging with 10 or 30 ng plasmid, respectively.

### Live cell imaging

Cells grown on eight-well slides (Ibidi) were cultured in complete DMEM and subjected to a double thymidine block. About 24 h prior to imaging, the expression of indicated constructs was induced using 1-2 ng ml^−1^ doxycycline. Two hours after release from the second thymidine block, the medium was changed to L-15 medium (Life Technologies) supplemented with 10% foetal bovine serum (Hyclone). The slide was mounted onto a Delta Vision Elite microscope (GE Healthcare) and cells were filmed for 16–24 h in 6–20-min intervals using a × 40, 1.35 numerical aperture, WD 0.10 objective. All data analysis was performed using the softWoRx software (GE Healthcare).

### Purification of interphase APC/C–Cdc20

Cdc20 complexes were purified from cells 8 h after release from a 20-h thymidine block and harvested by trypsin treatment after thorough washing with PBS. About 24 h (cells expressing YFP, YFP–Cdc20^WT^ and YFP–Cdc20^Ala^) or 8 h (cells expressing YFP–Cdc20^Asp^) prior to harvest, the expression of the indicated constructs was induced using 1–2 ng ml^−1^ doxycycline. Cells were lysed in lysis buffer (150 mM NaCl, 50 mM Tris-HCl pH 7.8, 1 mM DTT, 0.1–1% NP40 and 0.5 U μl^−1^ Benzonase) supplemented with PhosStop and Complete Mini (Roche) with a volume correlated to the cell pellets weight. Cdc20 complexes were immunoprecipitated without clearance of the lysate using a mouse monoclonal Cdc20 antibody cross-linked to Protein G-Sepharose 4B (Life Technologies) or YFP affinity resin (Chromotek) overnight at 4 °C. Precipitated protein complexes were washed in lysis buffer after precipitation of beads at 30*g* for 30 s and eluted in 4 × SDS sample buffer (Life Technologies). For Calyculin A (Cell Signalling) treatment, the drug was added for 30 min prior to harvest after 8 h at a concentration of 100 nM.

### Purification of BubR1 and APC3 to detect phosphorylation

Cells were harvested at indicated time points after a single or double thymidine block and collected by trypsin treatment after gentle washing with PBS. Cell pellets were washed with PBS and stored at −80 °C until further preparation.

Cells were lysed in lysis buffer (150 mM NaCl, 50 mM Tris-HCl pH 7.8, 1 mM DTT, 0.1–1% NP40). Lysis buffer was supplemented with PhosStop (not when samples were subject to **λ**PPase treatment) and Complete Mini. Samples were sonicated using a Bioruptor NextGen (diagenode). BubR1 and APC3 were immunoprecipitated using mouse monoclonal BubR1 or APC3 antibody cross-linked to Protein G-Sepharose 4B for 1 h at 4 °C. Precipitated protein complexes were washed in lysis buffer and eluted in 4 × SDS sample buffer.

### Drug treatment

HeLa cells were synchronized with double thymidine block released for 6 h and treated for 2 h with DMSO, 10 μM Cdk1/2 Inhibitor III (Millipore), RO3306 (Calbiochem) or 1 μM OA (Cell Signalling).

### Preparation of protein stability samples

Cells were released for 6 h from a 20-h thymidine block and treated with Cycloheximide (25 μg μl^−1^) and/or MG132 (10 μM) for 80 min. Cells were collected by trypsin treatment and the cell pellet was washed with PBS and samples were stored at −80 °C until further preparation.

### Quantitative infrared western

All cell lysates and immunoprecipitations were analysed by LI-COR quantitative infrared western technology. Proteins were separated by SDS–PAGE and blotted onto Immobilion FL membrane (Millipore). Membranes were incubated with indicated primary antibody and subsequently with IRDye 800 or 680 secondary antibodies (LI-COR). Membranes were scanned using the Odyssey Sa imaging system (LI-COR) and quantification was carried out using the Odyssey Sa Application software (LI-COR). Uncropped scans of key western blots are found in Supplementary Fig. 3.

## Additional information

**How to cite this article:** Hein, J. B. & Nilsson. J. Interphase APC/C–Cdc20 inhibition by cyclin A2–Cdk2 ensures efficient mitotic entry. *Nat. Commun.* 7:10975 doi: 10.1038/ncomms10975 (2016).

## Supplementary Material

Supplementary Information Supplementary Figures 1-3 and Supplementary Methods

Supplementary Movie 1 Mitotic entry in YFP-Cdc20^WT^ expressing cells monitored by time-lapse microscopy after release from a thymidine block.

Supplementary Movie 2 Mitotic entry in YFP-Cdc20^Asp^ expressing cells monitored by time-lapse microscopy after release from a thymidine block.

Supplementary Movie 3 Mitotic entry in YFP-Cdc20^Ala^ expressing cells monitored by time-lapse microscopy after release from a thymidine block.
