# Supplementary material for: Interphase APC/C–Cdc20 inhibition by cyclin A2–Cdk2 ensures efficient mitotic entry
Source: Nat Commun. 2016 Mar 10;7:10975. doi: 10.1038/ncomms10975 (PMC4792957; doi:10.1038/ncomms10975)
Supplement: Supplementary Information — Supplementary Figures 1-3 and Supplementary Methods [file ncomms10975-s1.pdf]

# SUPPLEMENTARY INFORMATION

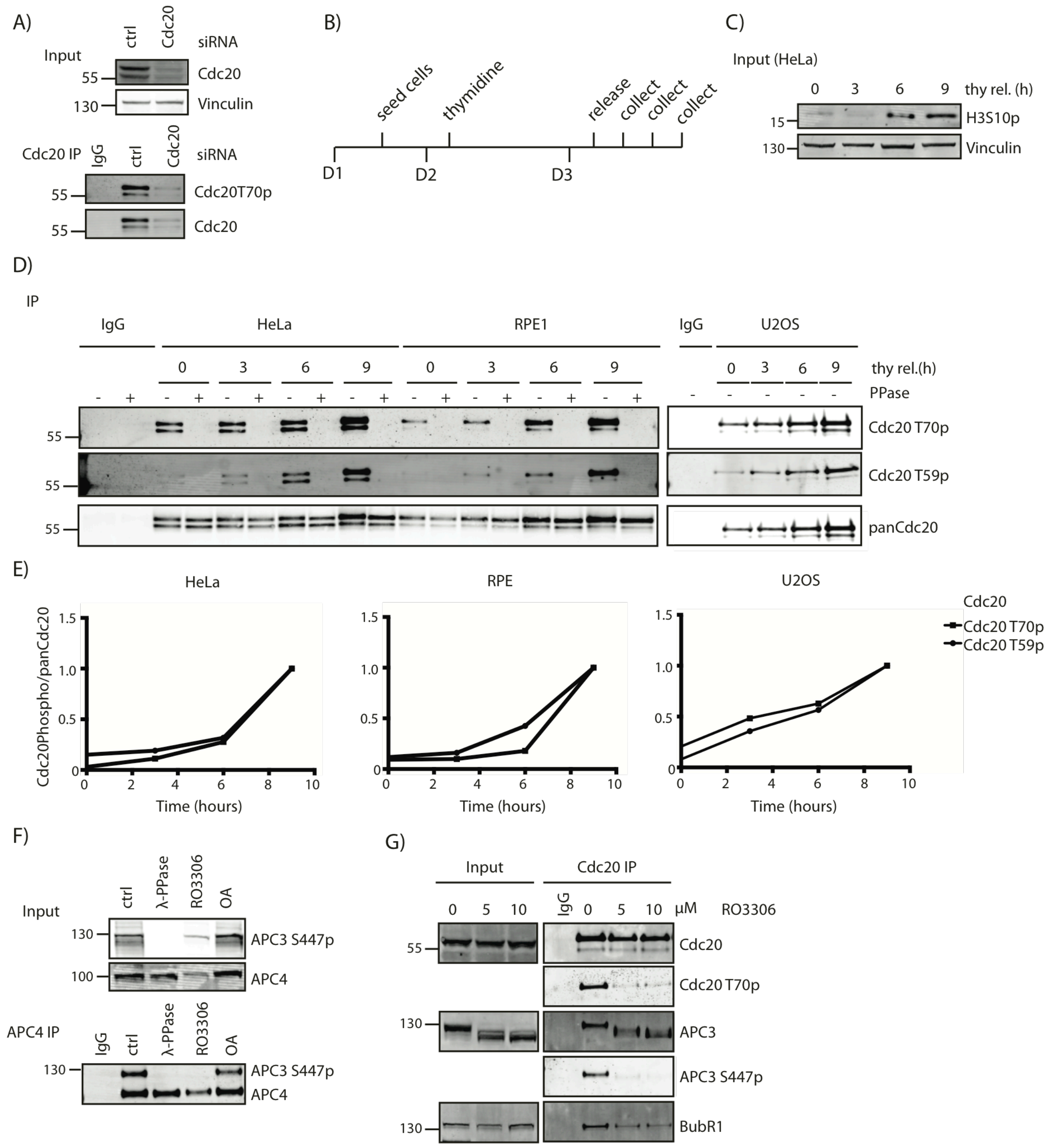


Supplementary Figure 1, Nilsson

**Supplementary Figure 1. Phosphorylation of Cdc20 in interphase**

**A)** Cdc20 was immunoprecipitated from HeLa cells treated with either an RNAi oligo against luciferase (ctrl) or Cdc20. The samples were analyzed by SDS-PAGE and western blot using a pan-Cdc20 antibody or an antibody recognizing the Cdc20 T70 phosphorylation. **B)** Outline of experimental setup to synchronize cells using a single thymidine block and then release. For time course experiments to analyze Cdc20 phosphorylation, cells were collected at different times after release from the thymidine block. **C)** Whole cell extract from experiments as in B), blotted for H3S10 phosphorylation to check synchronization protocol. **D)** The indicated cell lines were synchronized as outlined in B) and then Cdc20 was immunoprecipitated at the indicated time after release. Where indicated with +, the samples were treated with lambda phosphatase (λPPase). Samples were analyzed by SDS-PAGE and western blot using a pan specific Cdc20 antibody or the Cdc20 T70 or T59 phospho-specific antibodies. **E)** Quantification of the signal obtained from the phospho-specific antibodies normalized to the pan Cdc20 signal. The maximum phosphorylation level (9 hours after thymidine release) for each cell line is set to one. **F)** HeLa cells arrested with nocodazole where collected by shake-off and reseeded into media with DMSO, RO3306 (RO) or okadaic acid (OA). The cells were collected and cell extract from a DMSO treated sample was treated with λ−PPase. The APC/C complex was immunopurified with an anti-APC4 antibody and samples analyzed by SDS-PAGE and western blot for APC4 and APC3 S447p. **G)** HeLa cells were synchronized by a 24 hour thymidine block, release for 16 hours into nocodozole and collected by mitotic shake off and reseeded in media containing nocodazole with DMSO or indicated concentrations of RO3306 for 20 minutes. Cells were collected and Cdc20 immunopurified  and analyzed by SDS-PAGE and western blot.

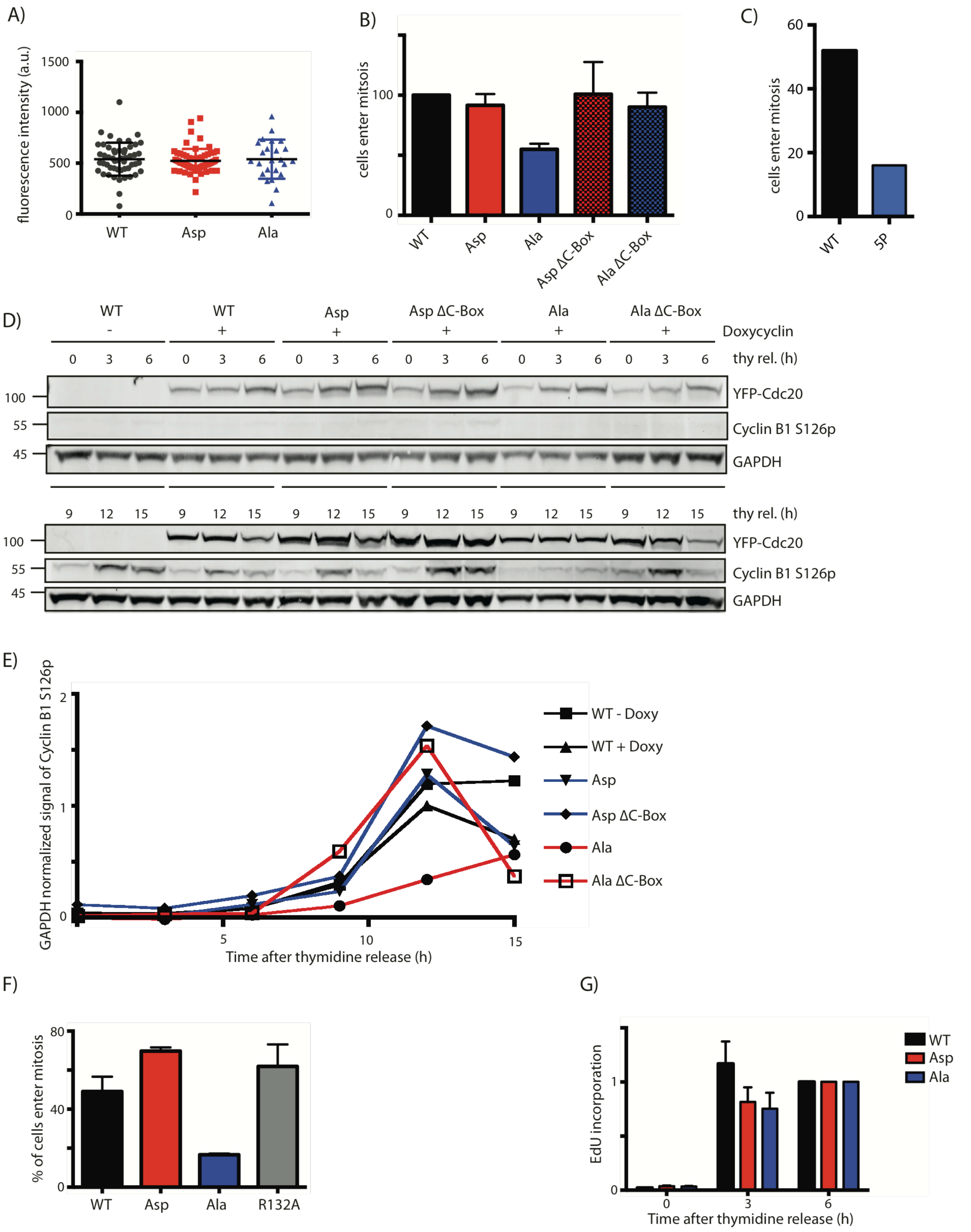


Supplementary Figure 2, Nilsson

**Supplementary Figure 2. Characterization of mitotic entry defects**

**A)** The fluorescence intensity of YFP-tagged Cdc20 proteins in the HeLa cells analyzed in the experiment shown in Figure 3B-C. The total fluorescence of single cells was measured and each point marks a single cell analyzed. The average and standard deviation is indicated. **B)** Stable inducible U2OS cells expressing the indicated YFP-tagged Cdc20 proteins were synchronized using a thymidine block and depleted of endogenous Cdc20. After release from the thymidine block cells were followed by live cell microscopy and cells entering mitosis were counted. The median and range from two independent experiments (at least 120 cells) is indicated. **C)** Stable HeLa-FRT cells expressing either YFP-Cdc20 WT or 5P (proline 42, 56, 60, 71 and 107 mutated to alanine) were synchronized by a double thymidine block, depleted of endogenous Cdc20 by siRNA and followed by time-lapse microscopy. Cells entering mitosis were counted (at least 175 per condition). **D)** Stable HeLa cell lines expressing the indicated YFP-tagged Cdc20 proteins were synchronized using a thymidine block and depleted of endogenous Cdc20. Where indicated the exogenous YFP-tagged Cdc20 protein was induced with doxycycline (+). After release from the thymidine block all cells were collected at the indicated time and the total extract analysed by SDS-PAGE and western blot for YFP-Cdc20 and Cyclin B1 S126p. **E)** Quantification of experiment as in D). Relative fluorescence intensity of Cyclin B1 S126 phosphorylation normalized to GAPDH with YFP-Cdc20$^{WT}$ expressing cells set to one. Representative of at least three independent experiments. **F)** Stable HeLa cells expressing the indicated YFP-tagged Cdc20 proteins were synchronized using two thymidine blocks and depleted of endogenous Cdc20. After release from the last thymidine block cells were monitored by live cell microscopy and the number of cells entering mitosis counted and normalized to the total number of cells analysed. Graph shows median with range of two independent experiments (at least 360 cells were analysed). **G)** Stable HeLa

cells expressing the indicated YFP-tagged Cdc20 proteins were synchronized using a thymidine block. At the indicated time after release cells were pulse labelled with EdU for 20 minutes, fixed and stained with DAPI and a fluorescently labelled antibody against EdU. Microscopy images were obtained and analysed using Olympus ScanR and ScanR analysis software that automatically detects the nucleus and measures the intensity of EdU staining. The bar indicates the median and range from two independent experiments after normalization to the six-hour time point.

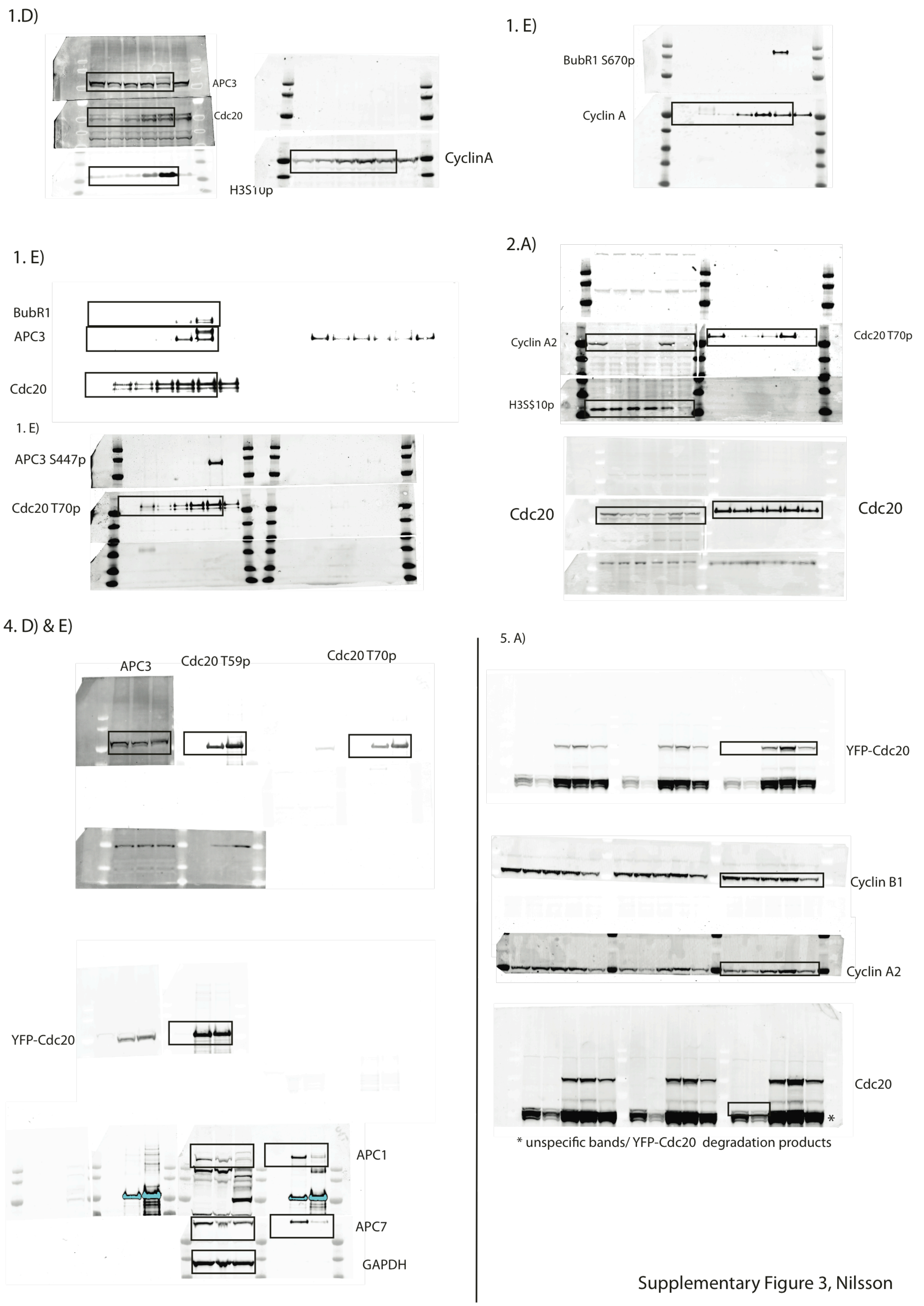


**Supplementary Figure 3. Uncropped western blots.**

Western blots used for main figures as indicated. Black boxes indicate cropped parts.

## SUPPLEMENTARY METHODS

### EdU incorporation

Indicated stable HeLa-FRT cells were seeded in a 24-well imaging plate, depleted of endogenous Cdc20 synchronized with thymidine released for 6 and 3 hours and EdU labeled for 20 minutes with Click-iT EdU Imaging Kit (Molecular Probes, Thermo Fisher) according to manufactures instructions. Plates were imaged using a ScanR imaging system (Olympus) with an Olympus IX-81 wide-field microscope with an MT20 illumination system and a digital monochrome Mahatsu C9100 CCD camera. Olympus UPLSAPO 20x/0.75 NA objective was used. Image analysis was caries out using the ScanR acquisition software.
